# The integrative role of orexin/hypocretin neurons in nociceptive perception and analgesic regulation

**DOI:** 10.1038/srep29480

**Published:** 2016-07-07

**Authors:** Ayumu Inutsuka, Akira Yamashita, Srikanta Chowdhury, Junichi Nakai, Masamichi Ohkura, Toru Taguchi, Akihiro Yamanaka

**Affiliations:** 1 Department of Neuroscience II, Research Institute of Environmental Medicine, Nagoya University, Nagoya, 464-8601 Japan; 2 Saitama University Graduate School of Science and Engineering, 255 Shimo-Okubo, Sakura-ku, Saitama City, Saitama, 338-8570 Japan; 3 Saitama University Brain Science Institute, 255 Shimo-Okubo, Sakura-ku, Saitama City, Saitama, 338-8570 Japan

## Abstract

The level of wakefulness is one of the major factors affecting nociception and pain. Stress-induced analgesia supports an animal’s survival via prompt defensive responses against predators or competitors. Previous studies have shown the pharmacological effects of orexin peptides on analgesia. However, orexin neurons contain not only orexin but also other co-transmitters such as dynorphin, neurotensin and glutamate. Thus, the physiological importance of orexin neuronal activity in nociception is unknown. Here we show that adult-stage selective ablation of orexin neurons enhances pain-related behaviors, while pharmacogenetic activation of orexin neurons induces analgesia. Additionally, we found correlative activation of orexin neurons during nociception using fiber photometry recordings of orexin neurons in conscious animals. These findings suggest an integrative role for orexin neurons in nociceptive perception and pain regulation.

Regulation of nociception is essential for survival in all animals. Lack of nociception and pain results in frequent injury without awareness^1^,^2^, while strong pain can be an obstacle to prompt responses such as fight or flight. It has been shown that pain is remarkably attenuated in soldiers during combat^3^ or in athletes during competition^4^. In these cases, the descending pain modulatory system regulates the strength of pain by sensing multiple factors such as stress, emotion and cognitive state^5^,^6^ in a context-dependent manner.

Orexin (hypocretin)-producing neurons (orexin neurons) in the hypothalamus are major regulators of wakefulness^7^,^8^. Two mature peptides, orexin A and orexin B, are derived from a precursor peptide, prepro-orexin. Orexin A and B are 46% homologous, and the sequence of orexin A is fully conserved across mouse, rat and human^9^. This fact strongly suggests the importance of these peptides for survival. A small number of orexin neurons are exclusively localized to the lateral hypothalamic area (LHA), but send their axons throughout the brain^10^,^11^ and even into the spinal cord^12^. As expected from this projection pattern, orexin neurons have multiple physiological functions not only in wakefulness but also in feeding^13^,^14^, reward^15^,^16^ and nociception^17^,^18^,^19^.

Central administration of orexin peptides reduces nociceptive responses in a mouse model of thermal, inflammatory and visceral pain^20^,^21^. In addition, orexin neurons innervate several brain regions related to nociception. The locus coeruleus (LC) is the main source of noradrenergic ascending and descending projections. Noradrenergic neurons in the LC predominantly express the orexin type 1 receptor (OX1R)^22^ and receive the most dense innervation from orexin neurons^23^. Recent studies using optogenetics clearly showed the functional importance of neural pathways from orexin neurons to the noradrenergic neurons in the LC during the sleep-wakefulness transition^24^,^25^. The periaqueductal gray (PAG) is also heavily innervated by orexin neurons, and OX1R in the PAG is suggested to be involved in anti-nociception^17^. These findings suggest that orexin peptides play pivotal roles in nociception.

Although the function of orexin peptides have been extensively studied, the physiological role of orexin neuronal activity in nociception remains unclear. Orexin neurons contain and release several neurotransmitters in addition to orexin, other neuropeptides and chemical neurotransmitters, such as dynorphin^26^, neurotensin^27^ and glutamate^28^. Dynorphin is an endogenous ligand of kappa opioid receptor^29^ and has strong analgesic effects^30^. Therefore, other neurotransmitters besides orexin might be involved in the regulation of nociceptive processing in a reciprocal or synergistic manner. Thus, it is worth investigating how the activity of orexin neurons affects nociceptive processing and pain-related behaviors in animals. In order to study this, selective recording and manipulation of the activity of orexin neurons is essential.

In this study, we utilized pharmacogenetic tools and temporally controlled ablation of orexin neurons to investigate the physiological roles of orexin neurons in nociceptive processing. Furthermore, we employed a fiber photometry system to achieve real-time measurement of the activity of orexin neurons during pain perception in conscious and anesthetized mice. Our results show a clear correlation of orexin neuronal activity and pain perception, and reveal a role for orexin neurons in the direct linkage between nociception and analgesia.

## Results

### Induction of selective ablation of orexin neurons and their co-transmitters in adult mice

**Figure 1:**
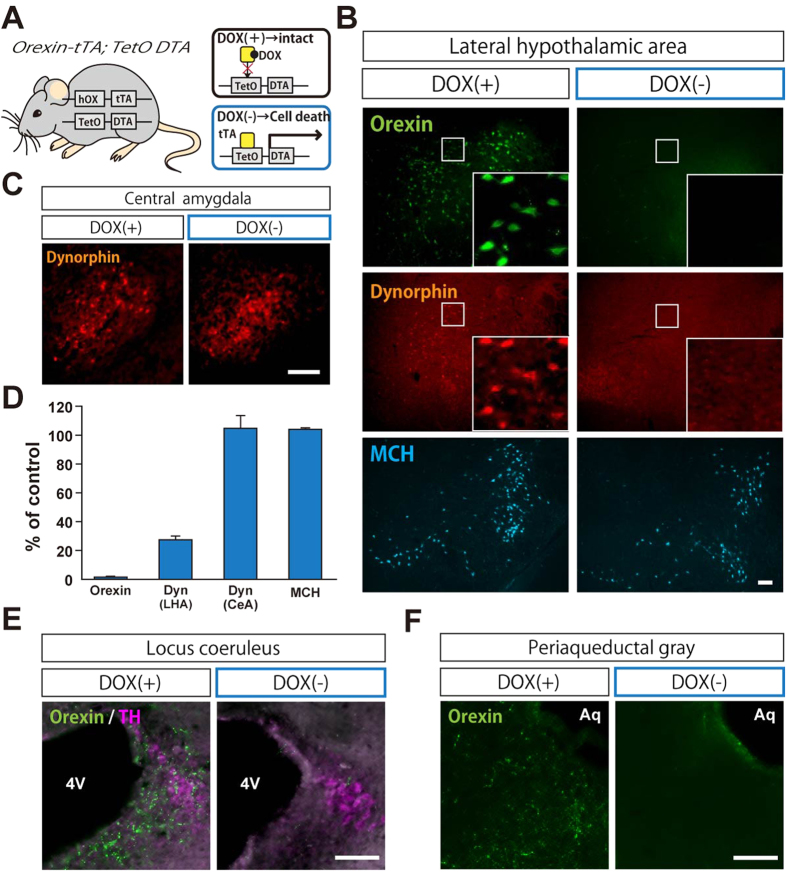
Selective ablation of orexin neurons in adult *orexin-tTA; TetO DTA* mice. (**A**) Schematic drawing showing the temporally-controlled specific ablation of orexin neurons using the tet-off system in *orexin-tTA; TetO DTA* mice. In the absence of DOX, orexin neurons are specifically ablated by expressing DTA. (**B**,**C**) Immunohistochemical confirmation of DOX-controlled specific ablation of orexin neurons. Coronal sections of the LHA (**B**) and the CeA (**C**) of *orexin-tTA; TetO DTA* mice fed with chow including DOX (DOX(+)) or chow without DOX (DOX(−)) for 4 weeks. Insets in (**B**) are magnifications of the boxed areas in each panel. (**D**) Quantitative analysis of the number of orexin-, dynorphin- and MCH-immunoreactive neurons in DOX(−) mice. Each number was normalized using the mean of DOX(+). Data represent the mean ± SEM (*n* = 3). (**E**,**F**) Coronal sections of the LC (**E**) and PAG (**F**). These areas include dense nerve endings of orexin neurons in DOX(+) mice. However, these orexin nerve endings were not observed in DOX(−) mice. 4V: fourth ventricle, Aq: Aqueduct, TH: tyrosine hydroxylase.

To investigate the role of orexin neurons in nociception, we selectively ablated orexin neurons in adult mice using the tet-off system. This system enables temporally-controlled induction of selective cell death. In *orexin-tetracycline transactivator (tTA); TetO diphtheria toxin A fragment (DTA)* mice, tTA is exclusively expressed in orexin neurons, and tTA binds to the TetO sequence. This eventually causes selective cell death of orexin neurons via DTA expression. Binding of tTA protein to the TetO sequence is blocked in the presence of doxycycline (DOX), which is supplied through the diet. Thus this neuronal ablation can be prevented during the developmental period by simply feeding the mice with chow containing DOX (100 mg/kg, DOX(+)). Orexin neuron-specific ablation is initiated by replacing DOX containing chow with normal chow (Fig. 1A).

Our previous study showed that a DOX(−) diet for 4 weeks ablated more than 95% of orexin neurons^31^. To confirm this, 4 weeks after switching from DOX(+) to DOX(−) chow, we sacrificed *orexin-tTA; TetO DTA* mice and counted the number of orexin neurons by immunohistochemistry. In the LHA of control mice fed DOX(+) chow, many orexin-immunoreactive (ir) neurons were observed. However, 98.7 ± 0.6% (n = 3) of orexin neurons were ablated in mice fed DOX(−) chow compared with control mice kept on a DOX(+) diet. The number of melanin concentrating hormone-producing neurons (MCH neurons), which are located near the orexin neurons in the LHA, was not affected (104.1 ± 1.1%, n = 3) compared to control mice (Fig. 1B,D). In accordance with the loss of orexin peptides in the LHA, we confirmed a large decrease in orexin nerve endings in the PAG, the LC, and the spinal cord in DOX(−) mice which are thought to be involved in nociception; dense projections of orexin neuronal axons were also observed in DOX(+) mice (Fig. 1E,F and Supplementary Fig. 1).

It has been reported that dynorphin is co-localized with orexin in the LHA^26^. Consistent with this, we observed a large decrease in dynorphin-ir neurons in the LHA of DOX(−) mice (Fig. 1B). 72.6 ± 2.5% (n = 3) of dynorphin-ir neurons were missing in the LHA compared with DOX(+) mice. This loss of dynorphin-ir neurons was selective to the LHA region as the number of dynorphin-ir cells in other areas was almost unchanged, e.g. in the central amygdala (104.8 ± 12.9%, n = 3) compared with DOX(+) mice (Fig. 1C,D and Supplementary Fig. 1). These results confirmed the temporally-controlled and selective ablation of orexin neurons and their co-transmitters by removal of DOX in *orexin-tTA; TetO DTA* mice.

### Enhanced pain-related behaviors with selective ablation of orexin neurons

**Figure 2:**
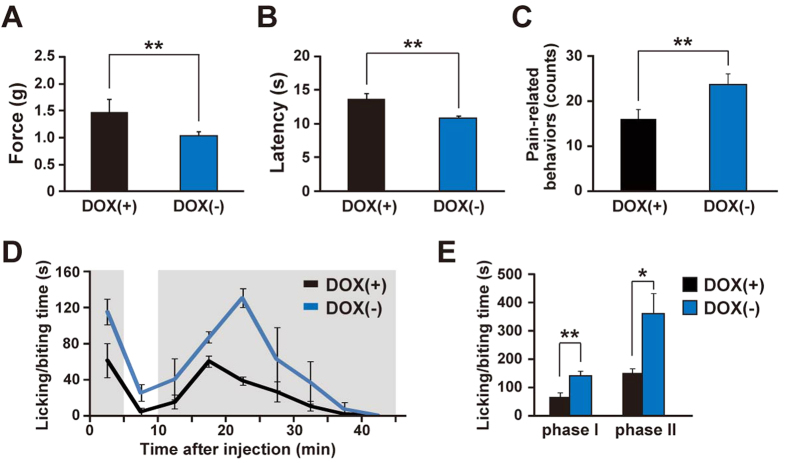
Enhanced pain-related behaviors in orexin neuron-ablated mice. (**A**) Von Frey tests for mechanical nociception (DOX(+): *n* = 16, DOX(−): *n* = 20). Escape force was measured. (**B**) Hargreaves tests for heat nociception (DOX(+): *n* = 12, DOX(−): *n* = 15). Escape latency was measured. (**C**) Cold plate tests for cold nociception (DOX(+): *n* = 12, DOX(−): *n* = 15). The number of pain related behaviors were counted. (**D**) Formalin tests for chemical nociception (DOX(+): *n* = 4, DOX(−): *n* = 5). The licking and biting time after formalin injection was measured. (**E**) Quantitative analysis of the formalin test; Phase I: 0–5 min, Phase II: 10–45 min. Data represent the mean ± SEM. Statistical analyses were performed by a Student’s t-test (*p < 0.05, **p < 0.01).

To investigate nociception in orexin neuron-ablated mice, these mice were subjected to several behavioral tests. In von Frey tests for mechanical nociception, DOX(−) mice showed a significant decrease in hind-paw withdrawal threshold force compared to DOX(+) control mice (DOX(+): 1.5 ± 0.3 g; DOX(−): 1.0 ± 0.1 g; n = 16, 20) (Fig. 2A). Similar nociceptive hypersensitivity was also observed for noxious heat and cold stimuli. DOX(−) mice showed a significant reduction in the latency of hind-paw withdrawal from the heat ray in the Hargreaves test (DOX(+): 13.6 ± 0.9 sec; DOX(−): 10.9 ± 0.3 sec; n = 12, 15) (Fig. 2B). In the cold plate test, a significant increase in pain-related behaviors was observed in DOX(−) mice (DOX(+): 16.0 ± 2.0 counts; DOX(−): 23.8 ± 2.2 counts; n = 12, 15) (Fig. 2C). We also performed a formalin test to analyze the time course for chemical pain. In the control DOX(+) mice, a subcutaneous injection of formalin into the hind-paw induced a clear biphasic display (phase I: 0–5 min, phase II: 10–45 min) of licking and biting of the injected hind-paw. DOX(−) mice showed similar but significantly longer licking and biting times in both phase I (DOX(+): 65 ± 16 sec; DOX(−): 140 ± 17 sec; n = 4, 5) and phase II (DOX(+): 151 ± 16 sec; DOX(−): 363 ± 70 sec; n = 4, 5) (Fig. 2D,E). Combined results from these tests for nociception indicated that ablation of orexin neurons increased pain perception generated by mechanical, thermal and chemical noxious stimuli.

### Attenuated pain-related behaviors by selective activation of orexin neurons

**Figure 3:**
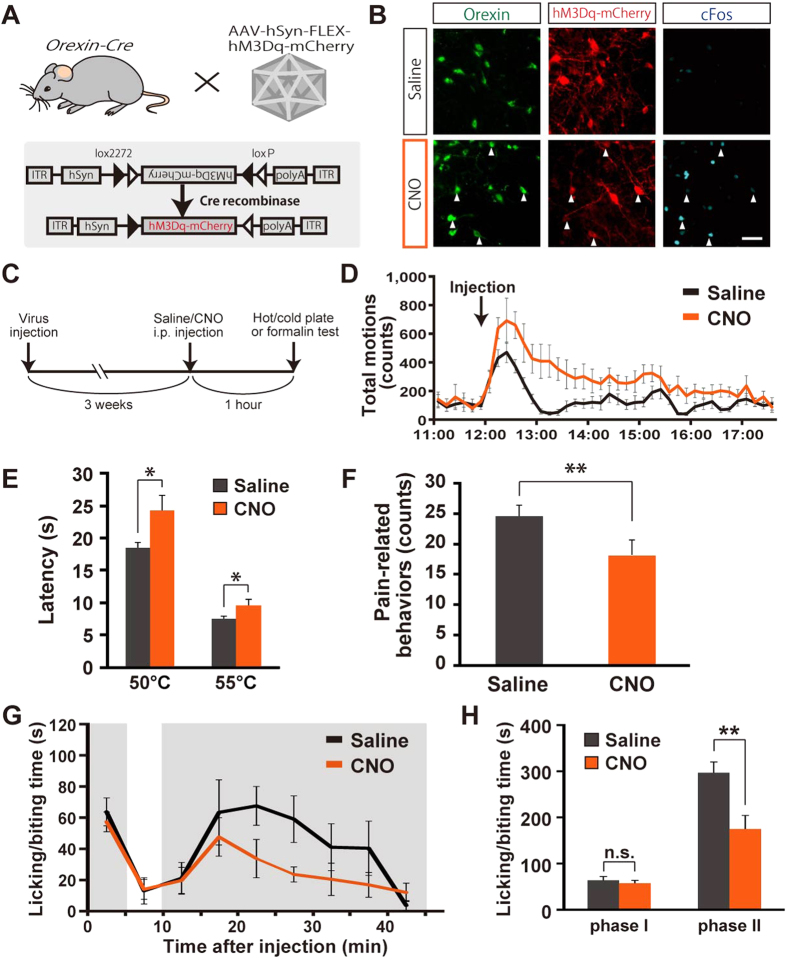
Attenuated pain-related behaviors in orexin neuron-activated mice. (**A**) Schematic drawing showing the specific expression of hM3Dq-mCherry in orexin neurons by bilaterally injecting AAV vectors expressing hM3Dq-mCherry in the presence of Cre recombinase into the hypothalamus of *orexin-Cre* mice. (**B**) Immunohistochemical confirmation of orexin neuron activation via hM3Dq. *Orexin-Cre* mice expressing hM3Dq in orexin neurons were intraperitoneally injected with saline or CNO (1.0 mg/kg). The arrowheads indicate triple co-localization of orexin, hM3Dq-mCherry and cFos. Scale bar is 50 μm. (**C**) Diagram illustrating the behavioral test protocol. AAV vectors were injected 3 weeks before the behavioral experiment day. On the day of experimentation, saline or CNO were intraperitoneally injected at 12:00 noon. One hour after injection, nociception assays were performed. (**D**) Locomotive activity was monitored to confirm the long lasting effects of orexin neuronal activation using pharmacogenetics. Total motion was counted every 10 min (saline: *n* = 12, CNO: *n* = 12). (**E**) Hot plate tests (saline: *n* = 12, CNO: *n* = 12). Latency to jump or licking was measured. (**F**) Cold plate tests (saline: *n* = 12, CNO: *n* = 12). The number of pain-rerated behaviors was counted. Data represent the mean ± SEM. Statistical analyses were performed by a Student’s t-test (*p < 0.05, **p < 0.01). (**G**) Formalin tests for chemical nociception (Saline: *n* = 6, CNO: *n* = 7). The licking and biting time after formalin injection was measured. (**H**) Quantitative analysis of the formalin test; Phase I: 0–5 min, Phase II: 10–45 min. Data represent the mean ± SEM. Statistical analyses were performed by a Student’s t-test (*n.s.* not significant, *p < 0.05, **p < 0.01).

Our findings that a lack of orexin neurons augments pain-related behaviors prompted us to test whether selective activation of orexin neurons would affect nociceptive processing and pain-related behaviors. To this end, we employed a pharmacogenetic tool, designer receptors exclusively activated by designer drugs (DREADD)^32^, as shown in Fig. 3A. In order to express the hM3Dq receptor exclusively in orexin neurons, a Cre-dependent adeno-associated virus (AAV) vector, AAV-hSyn-FLEX-hM3Dq-mCherry, was injected into the hypothalamus of *orexin-Cre* transgenic mice in which orexin neurons exclusively expressed Cre recombinase under control of human prepro-orexin promoter^13^. The specific expression and activation of orexin neurons was confirmed by immunohistochemistry. More than 3 weeks after injection of the AAV vector, saline or clozapine-N-oxide (CNO) (1 mg/kg) was intraperitoneally administered. One hour after application, cFos expression in orexin neurons was tested using immunohistochemical analysis. hM3Dq expression was detected as red fluorescence from the mCherry protein fused with hM3Dq. Although hM3Dq was expressed in orexin neurons in both groups, strong cFos expression was only observed in orexin neurons in the CNO-injected group (Fig. 3B, also refer to our previous report^13^). Behavioral experiments to test nociception were performed one hour after CNO injection (1 mg/kg, intraperitoneal (i.p.)) when orexin neurons were robustly and specifically activated (Fig. 3C). Locomotor assessment confirmed that there was an increase in locomotion which lasted for 4 hours (Fig. 3D) as in our previous study^13^. This hyper-locomotion made it difficult to perform the von Frey test or Hargreaves test. Therefore, we employed a hot plate test and a cold plate test. The temperature of the hot plate was set at 50 °C or 55 °C. The latency to exhibit pain-related behaviors such as hind-paw shaking or jumping was measured. The cut-off time was set at 1 min to avoid burn injuries. The cold plate test was performed as described in the methods. Compared with saline-injected mice, CNO-injected mice showed a significantly longer latency during the hot plate test at both 50 °C (Saline: 18.5 ± 0.7 sec, CNO: 24.3 ± 2.3 sec, n = 12) and 55 °C (Saline: 7.5 ± 0.4 sec, CNO: 9.5 ± 1.0 sec, n = 12) (Fig. 3E). Similar anti-nociceptive effects were observed in the cold plate test. Pain related behaviors were significantly decreased in CNO-injected mice (Saline: 24.4 ± 1.7 counts, CNO: 18.1 ± 2.6 counts, n = 12) (Fig. 3F). We also performed a formalin test to analyze the time course for chemical nociception. CNO-injected mice showed significantly shorter licking and biting time in phase II (Saline: 297 ± 23 sec, CNO: 175 ± 29 sec, n = 6, 7), while the initial response was not affected in phase I (Saline: 63.8 ± 8.2 sec, CNO: 57.4 ± 6.3 sec, n = 6, 7) (Fig. 3G,H). These results demonstrated that activation of orexin neurons using DREADD had an analgesic effect.

### Selective recording of orexin neuronal activity *in vitro*

**Figure 4:**
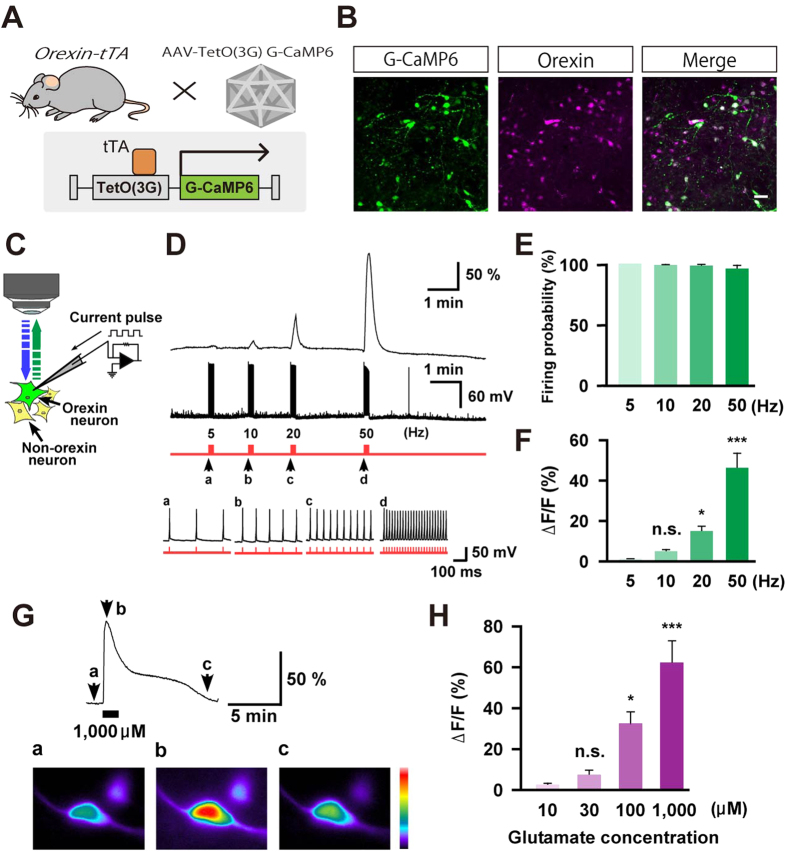
Calcium imaging to measure the activity of orexin neurons in brain slices. (**A**) Schematic drawing of G-CaMP6 specific expression in orexin neurons by injecting an AAV-TetO(3G) G-CaMP6 that expresses G-CaMP6 in the presence of tTA into the hypothalamus of *orexin-tTA* mice. (**B**) Immunohistochemical confirmation of specific expression of G-CaMP6 in orexin neurons. G-CaMP6 was identified by its own fluorescence. Scale bar is 50 μm. (**C**) Schematic drawing showing simultaneous recording of membrane potential by electrophysiology and intracellular calcium concentration changes by calcium imaging from an orexin neuron expressing G-CaMP6. (**D**) Representative traces showing the correlation between the action potential frequency (middle trace) and the increase in calcium concentration (upper trace). Action potentials were generated by injecting positive current (lower red trace) through the recording pipette (~50 pA) at 5 Hz (a), 10 Hz (b), 20 Hz (c), and 50 Hz (d), while the intensity of calcium fluorescence (ΔF/F) was simultaneously measured from the same orexin neuron. Arrows denoted by a, b, c, and d are expanded in the lower panel to show the faithful firing of orexin neurons. The upper trace indicates membrane potential (black) and the lower trace indicates the injected currents (red). (**E**,**F**) Summarized data showing induced firing probability (**E**) and ΔF/F (**F**) of orexin neurons from the experiment in (**D)** (*n* = 9). (**G**) Glutamate-induced activation of orexin neurons. Upper panel is a representative trace of calcium concentration when 1,000 μM of glutamate was applied. The lower panel shows pseudo-colored images of fluorescence intensity at different time points indicated by the arrows (a, b and c). (**H**) Data summarizing the ΔF/F of orexin neurons upon applying glutamate at different concentrations (*n* = 10–12). Values in (**E**,**F**,**H**) represent the mean ± SEM. All statistical analyses were made using Tukey’s multiple comparison test. (*n.s.* not significant, *p < 0.05, ***p < 0.001).

To elucidate the causality between neuronal activity and analgesic behaviors, it is necessary to perform real-time recordings of orexin neuronal activity during nociceptive processing. To achieve this, we first developed a recording system to detect selective responses of orexin neurons *in vitro* using a genetically encoded calcium indicator, G-CaMP6^33^. To express G-CaMP6 exclusively in orexin neurons, an AAV vector, AAV-TetO(3G) G-CaMP6, was injected into the LHA of *orexin-tTA* mice (Fig. 4A). The TetO(3G) sequence is a modified version of the TetO sequence that provides for lower basal expression leakage and higher expression in the presence of tTA^34^. Three weeks after AAV vector injection, we confirmed a dense overlap of the G-CaMP6 expression in orexin neurons (71.6 ± 7.4% of orexin neurons expressed G-CaMP6, n = 3, Fig. 4B). G-CaMP6 fluorescence was restricted to orexin neurons, and there was no ectopic expression of G-CaMP6 other than in orexin neurons. We prepared brain slices which included the LHA and performed simultaneous patch-clamp recording and calcium imaging to clarify the relationship between the electrophysiological properties and G-CaMP6 fluorescence (Fig. 4C). Representative traces indicated that orexin neurons expressing G-CaMP6 showed increased fluorescence intensity with firing in a frequency-dependent manner. Firing frequency was generated by injecting current pulse through a recording pipette at 5, 10, 20 and 50 Hz (Fig. 4D–F). We confirmed that orexin neurons responded to the input current with high fidelity even at a high 50 Hz frequency (Fig. 4D). The fluorescence of G-CaMP6 was enhanced as the frequency of firing increased (∆F/F% was 46.1 ± 7.4% when the applied firing was 50 Hz, n = 9, Fig. 4F). We also confirmed the calcium response of orexin neurons caused by glutamate bath application. By increasing the concentration of glutamate from 10 μM to 1,000 μM, we observed a correlative increase in fluorescence from 2.4 ± 0.8% to 62.4 ± 10.6% (n = 10–12, Fig. 4G,H). These results confirmed the robust correlation between G-CaMP6 fluorescence and activity of orexin neurons.

### Fiber photometry system for selective recording of orexin neuronal activity *in vivo*

**Figure 5:**
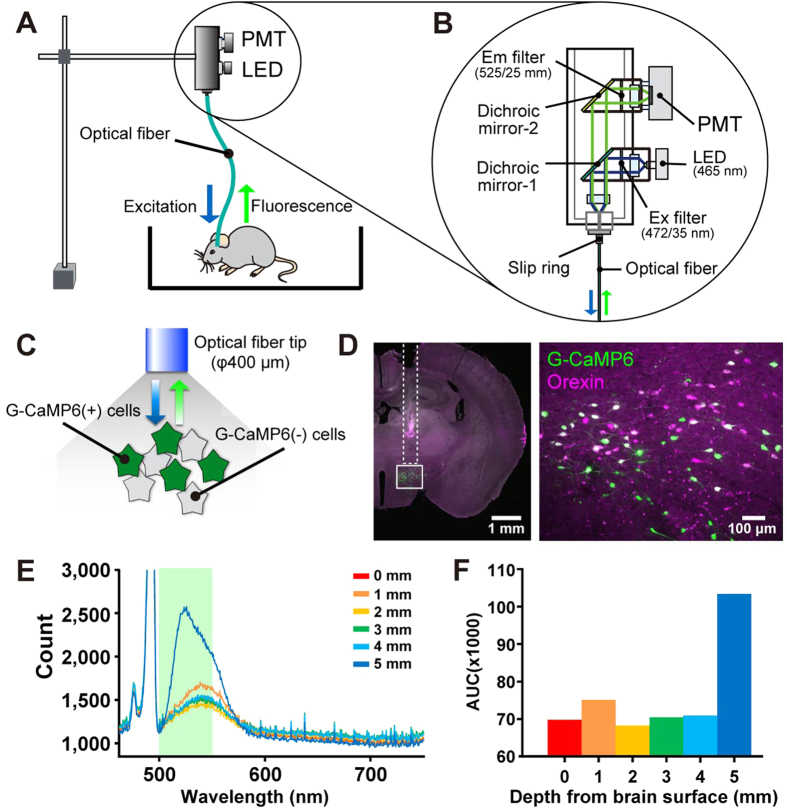
*In vivo* measurement of orexin neuron activity using fiber photometry. (**A–C)** Schematic drawings showing the apparatus for *in vivo* fiber photometry. (**A**) Overall view of the fiber photometry system. (**B**) Detail of the optical arrangement of the fiber photometry system. (**B**) is an enlarged image of the circled area in (**A)**. (**C**) Optical measurement of the change in intracellular calcium concentration from G-CaMP6-expressing orexin neurons. (**D**) Confirmation of the fiber tract after fluorescent measurements. Coronal section of the brain from an *orexin-tTA* mouse injected with AAV-TetO(3G) G-CaMP6. The dashed line indicates the location of the inserted fiber. The right panel is a magnified image of the boxed region in the left panel. (**E**) Confirmation of G-CaMP6-derived green fluorescence (500–550 nm) originating from orexin neurons of head-fixed mice. The spectrum of fluorescence was measured at intervals 1 mm in depth from the surface of the brain. G-CaMP6-derived green fluorescence (500–550 nm, green bar) was increased at a depth of 5 mm where orexin neurons were densely distributed. (**F**) Fluorescence intensity at each depth is summarized as the Area Under the Curve (AUC).

To achieve real-time recordings of orexin neuronal activity *in vivo*, we developed a novel fiber photometry system. Figure 5A–C show schematic drawings of our fiber photometry system (also refer to the Methods). In short, the system is equipped with an LED as a light source and a photomultiplier tube (PMT) as a detector. Blue light emitted from the LED is reflected by a dichroic mirror and illuminates the G-CaMP6-expressing neurons in the brain. The green fluorescence from G-CaMP6 is then gathered by the same optic fiber and the green light passes the first dichroic mirror and reflects off the second dichroic mirror in front of the PMT. Power intensity at the tip was set to 0.5 mW in the following experiments. To record the activity of orexin neurons, we generated mice which selectively express G-CaMP6 in orexin neurons by injecting AAV-TetO(3G) G-CaMP6 into the *orexin-tTA* mice as described in Fig. 4A. Three weeks after AAV vector injection, fiber photometric activity recordings of orexin neurons were performed. Figure 5E shows how we determined whether the recorded fluorescence originated from G-CaMP6 or not by analyzing the wavelength of the fluorescence. The fiber tip was gradually inserted into the brain surface and the wavelength of the fluorescence was measured at depths of 0, 1, 2, 3, 4 and 5 mm from the surface of brain. The location of the inserted optic fiber was confirmed histochemically after the experiments (Fig. 5D). At a depth of 5 mm, where orexin neurons are densely distributed, the 500–600 nm wavelength photon count greatly increased, and the peak shifted toward shorter wavelengths (Fig. 5E). This wavelength correlated well with the fluorescence from G-CaMP6^33^. The data summarized in Fig. 5F represent the area under the curve (AUC) between wavelengths at 500–550 nm that could pass through the fluorescence filter. These results confirmed that the fluorescent signals originated from G-CaMP6.

### Selective recording of orexin neuronal activity during nociception

**Figure 6:**
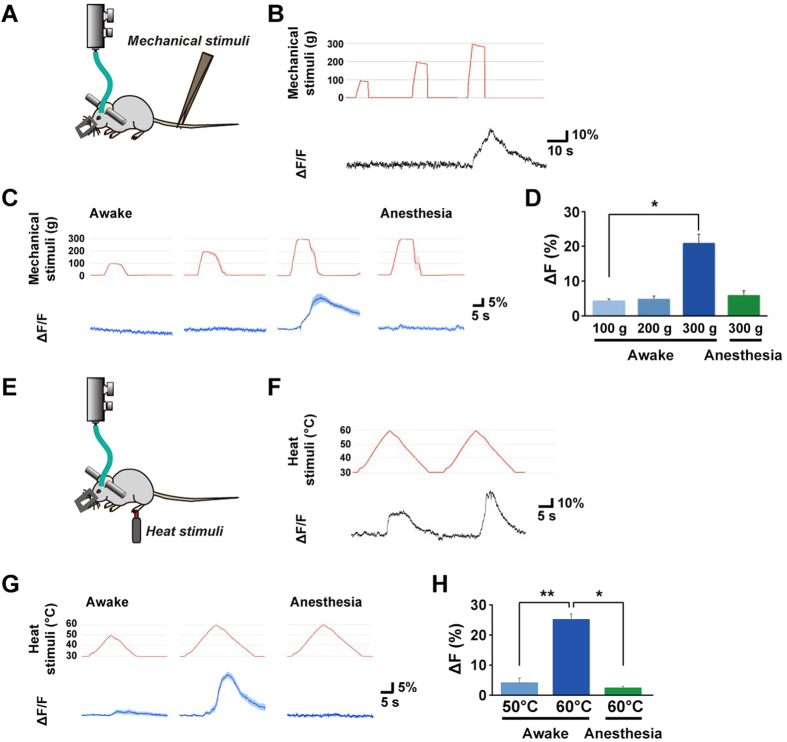
Activity recording of orexin neurons using fiber photometry during perception of noxious stimuli. (**A**) Mechanical noxious stimuli were applied to conscious or anesthetized mice. Mice were given mechanical stimulation (maximum strength: 100, 200 and 300 g) to the tail using forceps equipped with a force sensor at the tip. (**B**) Representative traces of fluorescence intensity changes evoked by mechanical stimulation in mice expressing G-CaMP6 exclusively in orexin neurons (**C**) Averaged mechanical stimulation (red) and fluorescence intensity change (blue) are shown as the mean ± SEM in awake and anesthetized mice (n = 4–6 mice). (**D**) % increases in fluorescence intensity induced by mechanical stimulation (*P < 0.05, one-way ANOVA followed by Dunn’s multiple comparison test). (**E**) Noxious heat stimuli were applied to conscious or anesthetized mice. Mice were given heat stimulation (maximum heat: 50 and 60 °C) to the plantar surface of the paw. (**F**) Representative traces of fluorescence intensity changes evoked by heat stimulation in mice expressing G-CaMP6 exclusively in orexin neurons. (**G**) Averaged heat stimulation (red) and fluorescence intensity change (blue) are shown as the mean ± SEM in awake and anesthetized mice (n = 5–7 mice). (**H**) % increase in fluorescence intensity induced by heat stimuli (*p < 0.05, **p *<* *0.01*, one-way ANOVA followed by Dunn’s multiple comparison test).

Using this fiber photometry system, we recorded the activity of orexin neurons in response to noxious stimuli under conscious and anesthetic conditions. Mice were head-fixed with a stereotaxic apparatus and an optic fiber was inserted (from bregma −1.5 mm, lateral 0.8 mm, ventral 5.0 mm); noxious mechanical stimuli were then applied to the tail using forceps equipped with a force sensor at the tip (Fig. 6A). Pinching force was programmable and was applied as a trapezoidal shape with 100, 200 and 300 g of maximum force. Before we use G-CaMP6-expressing mice, we confirmed that physical movement do not affect fluorescence intensity using *orexin-EGFP* mice as control (Fig. S2). The head position of a test mouse was stable even when mechanical stimuli induced body movement to avoid forceps. A representative trace indicates the activity of orexin neurons when 100, 200 and 300 g mechanical stimuli were applied (Fig. 6B). Pinching with a 300 g force induced a robust correlative increase in the G-CaMP6 signal of orexin neurons with high fidelity, while this response was not observed when weaker (100 g or 200 g) stimuli were applied or when the mice were under anesthesia with isoflurane (Fig. 6C,D).

We also investigated orexin neuronal activity during nociception of heat stimuli. A ramp-shaped noxious heat stimulus gradually increasing from 30 °C to 50 °C or 60 °C was applied to the left hind-paw using a thermal stimulator with a circular probe (diameter: 1 mm, Fig. 6E). Heat stimuli also induced a clear correlative increase in the fluorescent signal (Fig. 6F). A 50 °C stimulus induced a very weak response, however, at 60 °C a significant increase in activity of orexin neurons was induced. We also observed a temperature-dependent increase in the response that was not detected under anesthetic conditions (Fig. 6G,H). These results showed the correlative activation of orexin neurons during nociception.

## Discussion

In this study, we selectively manipulated the activity and fate of orexin neurons to elucidate their cellular contribution to nociceptive perception and pain regulation. Adult-stage ablation of orexin neurons, together with a considerable decrease in their co-transmitters in the LHA, demonstrated their roles in anti-nociception and analgesia to all modalities of painful stimuli such as mechanical, thermal, and chemical stimuli. Accordingly, pharmacogenetic activation of the same neurons attenuated thermally and chemically induced pain. Using our novel fiber photometry system, we found a robust correlation between orexin neuronal activity and nociceptive perception induced by mechanical and heat stimuli. Interestingly, these neuronal responses were not observed under inhalation anesthesia by isoflurane.

We found correlative activation of orexin neurons induced by noxious mechanical and heat stimuli. To our knowledge, this is the first study to demonstrate real-time recording of orexin neuronal activity in conscious mice during nociceptive processing. Previous studies have reported that the concentration of orexin peptides is relatively low when feeling pain based on microdialysis^35^, while c-Fos expression in orexin neurons increases after noxious stimuli such as carrageenan-induced inflammation or foot-shock^36^,^37^. However, the temporal resolution of these experimental techniques was insufficient to detect real-time changes in the activity of orexin neurons. In the present study, we carefully confirmed the relationship between the fluorescence of G-CaMP6 and electrical activity in orexin neurons even at a high frequency firing rate of 50 Hz, and developed a recording system for selective neural circuits in the deep brain. Our findings demonstrate the high sensitivity of the fiber photometry system for detection of transient fast activity during physiological responses. Recently, other groups have developed similar systems to reveal the precise activity pattern during feeding^38^, reward^39^ and social behavior^40^. These technical advances shall aid in dissecting the causality between specific neuronal activity and various behaviors. Our system can be further modified to add an optical path for photostimulation using different wavelengths of light. In the future, we expect that simultaneous recording and manipulation of orexin neurons in free-moving mice will characterize their multiple roles in nociception.

It is relatively easy to trace the efferent projections from orexin neurons. In contrast, the afferent pathways that convey noxious stimulation to the orexin neurons remain less clear. There are several candidate regions, however. The neurons in the spinal cord send axons directly to the hypothalamus^41^. The parabrachial nucleus is innervated by lamina I projection neurons in the spinal cord^42^ and also projects to orexin neurons^43^,^44^. Considering that the amygdala projects to orexin neurons^44^, it is reasonable to suspect their involvement in nociception, especially in the affective component of pain. Loss of orexin neurons in human beings results in the sleep disorder narcolepsy with a characteristic symptom called cataplexy^45^,^46^,^47^. Cataplexy is the sudden loss of muscle tone caused by strong emotion with a preservation of consciousness during the attack^48^. Furthermore, prepro-orexin knockout mice showed weaker cardiovascular and locomotor responses to emotional stress in awake and freely moving conditions^49^. The central amygdala receives multiple nociceptive information from the brainstem, as well as highly processed polymodal information from the thalamus and the cerebral cortex^50^. However, the amygdala and the parabrachial nucleus mediate quite varied physiological responses, therefore it is difficult to decipher specific functional pathway conveying nociceptive information in these areas.

The C1 neurons located in the rostral ventrolateral medulla are also interesting candidates. Comprehensive analysis using transgenic mice expressing a retrograde tracer, GFP-TTC, selectively in orexin neurons identified several brain regions including basal forebrain cholinergic neurons, serotonergic neurons in the raphe nucleus, and neurons in the ventrolateral medulla^51^. Moreover, it was confirmed that C1 neurons innervate the orexin neurons by immunohistochemistry and electron microscopy^52^. Considering that C1 neurons are activated by noxious stimuli and involved in autonomic responses^53^, orexin neurons might mediate the transmission of nociceptive information from the C1 neurons during nociception. Although activation of adrenaline receptors in orexin neurons inhibits neuronal activity^54^, the synapses formed by C1 neurons with orexin neurons are densely filled with small clear vesicles likely containing glutamate^52^. Indeed, pharmacogenetic activation of A1/C1 catecholamine neurons results in activation of orexin neurons^55^. To confirm this, retrograde trans-synaptic infection of recombinant rabies viral vectors in selective types of neurons^56^ might enrich our understanding of the possible inputs of orexin neurons, and selective manipulation technologies will confirm the physiological importance of those pathways.

Interestingly, orexin neurons could not be activated by noxious stimuli under anesthesia by isoflurane. It was reported that isoflurane and sevoflurane inhibit c-Fos expression in orexin neurons^57^, that selective ablation of orexin neurons delays the emergence from anesthesia^57^, and that orexin A facilitates emergence of the rat from isoflurane anesthesia^58^. These findings suggest the possibility that inhibition of orexin neurons by anesthetics plays a role in maintaining stable anesthesia.

Correlative activation of orexin neurons might affect memory formation by modulating the vigilance level of the entire brain. Noxious stimuli, such as foot-shock, are commonly used as unconditioned stimuli in associative learning. Immediate memory formation to avoid the succeeding experience is very important for survival. Indeed, orexin receptor 1 in the locus coeruleus is involved in fear memory consolidation^59^. It was also reported that orexin A has a beneficial inhibitory effect on orofacial pain-induced deficits in spatial learning ability and memory^60^. Our results show that noxious stimuli immediately activate orexin neuronal activity. The role of orexin neurons in linking nociception and cognition is an interesting topic for future study.

We found that temporally-controlled ablation of orexin neurons in adult mice results in enhanced pain-related behaviors against noxious mechanical, thermal and chemical stimuli. Previous studies have also shown the involvement of the orexin system in nociception. Intravenous injection of orexin A produced analgesic effects in a hot plate test and a carrageenan test^21^. Intra-PAG microinjection of orexin A decreased formalin-induced nociceptive behaviors^61^, and this analgesic effect is mediated by the orexin 1 receptor and endocannabinoid signaling^17^. Orexin neurons innervate the spinal cord^12^ (Fig. S1), and the spinal cord also expresses the orexin 1 receptor^62^. It has been reported that the spinal orexin 1 receptors mediate the anti-hyperalgesic effects of intrathecally-administered orexins in a rat model of diabetic neuropathic pain^63^. Our results enhance these findings and confirm the cellular role of orexin neurons in nociception. While prepro-orexin null mice show stress-induced analgesia, their baseline pain thresholds are the same as in wild type mice^36^. This behavioral difference between prepro-orexin null and *orexin-tTA; TetO DTA* mice might imply the concerted effect of co-transmitters within the orexin neurons. It was also reported that orexin neuron-ablation in transgenic mice using a neurotoxic protein, polyglutamine repeat of ataxin-3, resulted in stress-induced analgesia^64^. However, it should be noted that in the hot plate test, a significant difference appeared only after application of strain stress between wild type and *orexin-ataxin-3* mice. Our previous report showed that the sleep fragmentation and cataplexy phenotype in *orexin-tTA; TetO DTA* mice was much stronger than in *orexin-ataxin-3 mice*^31^. Compensation during development or higher ablation efficiency might explain the different experimental results between *orexin-tTA; TetO DTA* mice and *orexin-ataxin-3* mice. Our DREADD experiments revealed that activation of orexin neurons exerts analgesic effects for several hours. However, the initial time course in the formalin test was similar. This might suggest that i.p. injection itself activates endogenous analgesic mechanisms including orexin neurons. During the hot and cold plate tests, hyperactivity after CNO can be a confounding factor as the mice have less contact with the floor so there may be less heat transfer to the paw. The consistency between the results of neuronal activation and neurotransmitter administration is important because it is possible that the effects of excessive peptide administration do not reflect the physiological role of the peptide-containing neurons *in vivo*.

In addition, we observed a marked reduction of dynorphin-expressing neurons in the LHA by selective ablation of orexin neurons. Dynorphin plays critical roles in nociceptive processing^65^, and possibly acts with orexin to induce complex effects on downstream neurons. Although orexin and dynorphin exert opposing actions on motivated behavior^66^, the combined effect of these neuropeptides in nociception may be synergetic. One example of the discordant effect of these two peptides is that an opioid receptor antagonist did not inhibit the anti-nociceptive effects of orexin peptides^18^, while cocaine self-administration in OX1R KO mice was restored by kappa opioid receptor blockade^66^. Neurotensin is also expressed in orexin neurons and is known to regulate nociception^67^. Therefore, the analgesic effects caused by orexin neuronal activation might result from the combination of orexin peptides and other co-transmitters such as dynorphin or neurotensin. Considering that placebo analgesia is involved in the opioidergic descending pain inhibitory system^68^,^69^, the concerted action of orexin and dynorphin might be important for acute pain perception. Note that, similar to our previous report^13^, we did not employ colchicine treatment in our experiments, although many studies use colchicine to block axoplasmic transport and enhance the immunostaining signal of dynorphin in the soma.

Taken together, noxious stimuli induced correlated activation of orexin neurons, and pharmacogenetic activation of orexin neurons attenuated pain perception by heat and cold stimuli. Although it is difficult to determine from our experiments specific neuronal projection involving nociceptive processing, the use of sophisticated genetic tools that have recently been developed^39^ might help to dissect the complex physiological role of orexin neurons in the future. Our new findings suggest an integrative role for orexin neurons in nociceptive perception and analgesia, and highlight the linkage between nociception and wakefulness.

## Methods

### Animals

All experimental procedures involving animals were approved by the Institutional Animal Care and Use Committee of the Research Institute of Environmental Medicine, Nagoya University. All the *in vivo* experiments were performed in compliance with Nagoya University’s Animal Facility regulations. Mice were maintained under a strict 12 hour light/dark cycle (light period: 8:00–20:00; dark period: 20:00–8:00) in a temperature-controlled room (22 °C). Food and water were available ad libitum and all efforts were made to minimize animal suffering and discomfort and to reduce the number of animals used.

### Selective ablation of orexin neurons

*Orexin-tTA* mice, which express tetracycline transactivator (tTA) exclusively in orexin neurons under the control of human prepro-orexin promoter^70^, were bred with *TetO diphtheria toxin A fragment (DTA)* mice (B6.Cg-Tg(tetO DTA)1Gfi/J, The Jackson Laboratory) to generate *orexin-tTA; TetO DTA* mice^31^. In these double transgenic mice, DTA expression occurs in orexin neurons in the absence of doxycycline (DOX). Both *orexin-tTA* mice and *TetO DTA* mice were on the C57BL/6J genetic background. DOX-containing chow (DOX chow) was made by adding 10% DOX powder (Kyoritsu Seiyaku, Japan) to normal chow (MR stock, Nihon-Nosan, Japan) at a final concentration of 100 mg/kg. Mating pairs of *orexin-tTA* mice and *TetO DTA* mice were fed with DOX-containing chow (DOX(+) condition) from the day of mating. During the prenatal and early postnatal periods, DOX was supplied via maternal circulation or lactation, respectively. After weaning, *orexin-tTA; TetO DTA* mice were fed with DOX(+) chow until the day of the experiment. To start ablation, DOX(+) chow was replaced with MR stock (DOX(−) condition).

### Behavioral tests

#### von Frey test

Sensitivity to mechanical stimuli was tested using self-made von Frey filaments^71^. Mice were individually placed into plastic containers with perforated metal floors (Model 37450, Ugo Basile, Varese, Italy), and acclimated for at least 30 min before testing. A series of 10 filaments (bending forces 0.2~6.8 g in quasi-logarithmic order, 0.5 mm in diameter) was applied to the midplantar surface of the left hind-paw. We applied the weakest filament first, then each filament was applied twice at intervals of 1 min. The threshold was determined as the minimum force eliciting at least one clear withdrawal response of the paw within the two trials.

#### Hargreaves test

Thermal sensitivity to noxious heat was tested using a Hargreaves apparatus (Model 7371, Ugo Basile, Varese, Italy)^71^. Mice were placed into individual plastic cages with glass floors 30 min before the experiments. The withdrawal latency was measured in response to an infrared beam (intensity: IR40) applied to the plantar surface of the left hind-paw. The cut-off latency was set at 20 s to avoid damaging the tissue by heat.

#### Cold plate test

The nociceptive threshold to cold stimuli was measured by the cold plate test. Mice were placed on a cold stainless steel plate surrounded by clear Plexiglas (12 × 20 × 10 cm). The temperature of the cold plate was continuously monitored and kept at −5 °C by circulating water containing 20% ethylene glycol beneath the plate. Pain-related behaviors such as hind-paw lifting, licking and jumping were observed and videotaped. The total duration of the pain-related behaviors was measured during a 3 min observation period.

#### Formalin test

Following an acclimation period of at least 30 min, 10 μl of 5% formalin dissolved in 0.01 M PBS was injected subcutaneously into the plantar surface of the left hind-paw with a 30-gauge needle. Mice were returned to the plastic box immediately after the injection. The total duration of pain-related behaviors (i.e. licking and biting of the injected side hind-paw) were measured every 5 min up to 50 min after the injection. We identified and analyzed two phases in the pain-related behaviors: an initial acute phase (phase I, 0–5 min after the injection) followed by a prolonged tonic phase (phase II, 10–45 min after the injection).

### Adeno-associated virus (AAV) production and purification

All AAV vectors were produced using the AAV Helper-Free System (Agilent Technologies, Inc., Santa Clara, CA, USA) and purified according to published methods^72^. Briefly, HEK293 cells were transfected with a pAAV vector plasmid that included a gene of interest, pHelper and pAAV-RC (serotype DJ; purchased from Cell Biolabs Inc, San Diego, CA, USA) using a standard calcium phosphate method. Three days later, transfected cells were collected and suspended in artificial cerebrospinal fluid (aCSF; 124 mM NaCl, 3 mM KCl, 26 mM NaHCO_3_, 2 mM CaCl_2_, 1 mM MgSO_4_, 1.25 mM KH_2_PO_4_, and 10 mM D-Glucose). After 4 freeze-thaw cycles, the cell lysate was treated with benzonase nuclease (Merck, Darmstadt, Germany) at 45 °C for 15 min and centrifuged 2 times at 16,000 g for 10 min. The supernatant was used as the virus-containing solution. To measure the titer of purified virus, the supernatant was dissolved in artificial CSF. Quantitative PCR was performed to measure viral titer using the following primer pairs: woodchuck hepatitis virus posttranscriptional regulatory element (WPRE), WPRE-Forward: 5′-ACTGTGTTTGCTGACGCAAC-3′, WPRE-Reverse: 5′-CAACACCACGGAATTGTCAG-3′; human growth hormone polyA-Forward: 5′-TGGGAAGACAACCTGTAGGG-3′, human growth hormone polyA-Reverse: 5′-GTGAAACCCCGTCTCTACCA-3′. The AAV vector was stored at −80 °C in small aliquots until the day of experiment. The pAAV-hSyn-FLEX-hM3Dq-mCherry plasmid was purchased from Addgene (ID: 44361).

### Stereotaxic AAV injection

Surgeries for AAV injections were conducted under pentobarbital anesthesia (50 mg/kg, i.p.) and isoflurane (2%, inhalation) using a stereotaxic instrument (David Kopf Instruments, Tujunga, CA, USA). Recombinant AAV-hSyn-FLEX-hM3Dq-mCherry (serotype: DJ; 600 nl/injection, 3 × 10^12^ copies/ml) was stereotaxically and bilaterally injected into the LHA of *orexin-Cre* mice. A glass micropipette pulled with a puller (Sutter Instrument Novato, CA, USA) with a tip diameter of 100 μm was filled with AAV. An air pressure injector system (Pneumatic PicoPump; World Precision Instruments, Inc., Sarasota, FL, USA) was connected to the glass micropipette with a polyethylene tube. Air pressure (10–20 psi) was applied to inject the AAV. Injection sites were as follows: from bregma −1.4 mm, lateral ± 0.7 mm, ventral −5.0 mm for AAV-hSyn-FLEX-hM3Dq-mCherry. Three weeks after the AAV injection, mice were subjected to behavioral experiments.

### Immunohistochemistry

Mice were deeply anesthetized with isoflurane and transcardially perfused with 20 ml of chilled saline, followed by 20 ml of chilled 10% formalin solution (Wako Pure Chemical Industries, Ltd., Osaka, Japan). The brain was removed, post-fixed in 10% formalin solution at 4 °C overnight, and immersed in 30% sucrose in PBS at 4 °C for at least 2 days. A series of 40 μm sections were obtained with a cryostat (Leica CM3050 S; Leica Microsystems, Wetzlar, Germany).

For staining, coronal brain sections were immersed in blocking buffer (1% BSA and 0.25% Triton-X in PBS), then incubated with primary antibodies at 4 °C overnight. The sections were washed with blocking buffer then incubated with secondary antibodies for 1 hour at RT. The brain sections were mounted and examined with a fluorescence microscope (BZ-9000, Keyence, Osaka, Japan or IX71, Olympus, Tokyo, Japan). Primary antibodies and secondary antibodies were diluted in blocking buffer as follows: anti-orexin A goat antibody (Santa Cruz Biotechnology, Inc., Dallas, TX, USA) at 1:2000, anti-MCH rabbit antibody (Sigma-Aldrich, St. Louis, MO, USA) at 1:2000, anti-prodynorphin guinea pig antibody (EMD Millipore, Darmstadt, Germany) at 1:1000, CF488-conjugated anti-mouse or anti-rabbit antibody (Biotium Inc., Hayward, CA, USA) at 1:1000, CF594-conjugated anti-rabbit or anti-goat antibody (Biotium) at 1:1000 and CF647-conjugated anti-goat antibody (Biotium) at 1:1000. We did not use colchicine to enhance neuropeptide signal. We manually counted positive cells using NIH ImageJ software and calculated the relative percentage of positive neurons in DOX(−) mice by using the average number of positive neurons in DOX(+) mice as a reference. Numbers from absolute cell counts were as follows: orexin in DOX(+): 648 ± 47 cells, in DOX(−): 8 ± 4 cells, MCH in DOX(+): 1108 ± 9 cells, in DOX(−): 1153 ± 12 cells, dynorphin (LHA) in DOX(+): 495 ± 32 cells, in DOX(−): 135 ± 12 cells, dynorphin (CeA) in DOX(+): 189 ± 15 cells, in DOX(−): 198 ± 24 cells. For counting, we analyzed one out of every four coronal brain slices.

### Brain slice preparation and Electrophysiology

Both male and female *orexin-tTA* mice, 3–8 months old, were used for electrophysiological recordings and calcium imaging. These mice were injected with AAV-TetO(3G) G-CaMP6 (serotype: DJ; titer: 1 × 10^12^ copies/ml; volume: 1.2 μl/injection) in the LHA (from bregma −1.4 mm, lateral ± 0.8 mm, ventral −5.0 mm). At least 3 weeks post-injection, mice were deeply anesthetized using isoflurane and were decapitated. The brains were then quickly isolated and chilled in ice-cold cutting solution (in mM: 110 K-gluconate, 15 KCl, 0.05 EGTA, 5 HEPES, 26.2 NaHCO_3_, 25 Glucose, 3.3 MgCl_2_ and 0.0015 (±)-3-(2-Carboxypiperazin-4-yl)propyl-1-phosphonic acid) bubbled with 95% O_2_ and 5% CO_2_. Brains were cut into coronal sections at a thickness of 300 μm using a vibratome (VTA-1200S; Leica, Germany) and slices containing the LHA region were transferred to an incubation chamber containing bath solution (in mM: 124 NaCl, 3 KCl, 2 MgCl_2_, 2 CaCl_2_, 1.23 NaH_2_PO_4_, 26 NaHCO_3_ and 25 Glucose) and bubbled with 95% O_2_ and 5% CO_2_ at 35 °C in a water bath for 30 min. Brain slices were then incubated in the same chamber at room temperature for 30–60 minutes. Solutions were modified based on the method of Pressler *et al*.^73^.

Brain slices were transferred into a recording chamber (RC-27L; Warner Instruments, USA) on a fluorescence microscope stage (BX51WI; Olympus, Tokyo, Japan) and were superfused with bubbled (95% O_2_ and 5% CO_2_) bath solution at rate of 0.8 ml/min using a peristaltic pump (Dynamax, Rainin, USA). An infrared camera (C2741-79; Hamamatsu Photonics, Hamamatsu, Japan) was installed in the fluorescence microscope along with a charge-couple device camera (Evolve 512 delta; Photometrics, USA) and both images were separately displayed on a monitor. A micropipette puller (P-1000; Sutter Instruments) was used to prepare the patch pipette (GD150-10, Harvard Apparatus, USA) of 5–8 MΩ resistance. Patch pipettes were filled with a KCl-based internal solution (in mM: 145 KCl, 1 MgCl_2_, 10 HEPES, 1.1 EGTA, 2 MgATP, 0.5 Na_2_GTP; pH 7.3 with KOH) and the osmolarity of the solution was confirmed to be 280–290 mOsm. Orexin neurons were identified by green fluorescence of G-CaMP6. Positive pressure was introduced within the patch pipette and it was moved toward the cell. Upon contacting the cell, the pressure was released and a giga-seal was made. The patch membrane was ruptured by suction to form a whole-cell configuration. The membrane potential was monitored with an Axopatch 200B amplifier (Axon Instrument, USA). Orexin neurons were hyperpolarized by negative current injection around −70 to −80 mV through an amplifier to stop spontaneous firing. To generate faithful firing, a depolarizing rectangular current of ~50 pA, width of 10 msec, frequency of 5, 10, 20 and 50 Hz was applied using an electric stimulator (SEN-3301; Nihon Kohden, Japan) and was applied to the cell through the recording pipette. The output signals were low-pass filtered at 5 kHz and digitized at a 10 kHz sampling rate. Patch clamp data were recorded through an analog-to-digital converter (Digidata 1322A; Axon Instruments, Molecular devices, USA) using pClamp 10.2 software (Molecular Devices, USA).

### Calcium imaging

Brain slices were transferred into a recording chamber and orexin neurons were identified by green fluorescence of G-CaMP6^33^. Slices were superfused with bubbled (95% O_2_ and 5% CO_2_) bath solution at the rate of 0.8 ml/min. Excitation light for G-CaMP6 was emitted from a light source (Spectra light engine; Lumencor, Beaverton, OR, USA) controlled by Metamorph software (Molecular Devices, Sunnyvale, CA, USA). Light was guided to the microscope with a liquid light fiber with a diameter of 1 cm. Brain slices were illuminated with blue light of 475 ± 17.5 nm wavelength and 9.7 mW power through the objective lens of a fluorescence microscope. G-CaMP6 fluorescence intensity was recorded continuously using Metamorph software at a rate of 2 Hz with 100 msec of exposure time. To synchronize the calcium imaging and patch clamp recording, pClamp software was triggered by the TTL output from Metamorph software. Metamorph data were analyzed by setting the region of interest (ROI) on G-CaMP6 expressing orexin neurons and the ΔF/F was calculated from the average intensity of the ROI.

### *In vivo* recordings of neuronal activity using fiber photometry

A fiber photometry system (COME2-FTR/OPT, Lucir, Tsukuba, Japan) was used to record the activity of orexin neurons in conscious mice. This system utilizes a single silica fiber to deliver excitation light and detect fluorescence from G-CaMP6 simultaneously. Excitation blue light (465 nm, 0.5 mW at the tip of the silica fiber) was produced by a high-power LED system (PlexonBright OPT/LED Blue_TT_FC, Plexon, Dallas, TX, USA). The excitation blue light emitted from the LED was reflected by a dichroic mirror and coupled to a 400 μm silica fiber through an excitation bandpass filter (path 472 ± 35 nm). G-CaMP6 fluorescence was collected by the same silica fiber and guided to a photomultiplier (PMTH-S1M1-CR131, Zolix instruments, Beijing, China) passed through a bandpass emission filter (path 525 ± 25 nm). The signal was digitized using an A/D converter (Micro1401), and recorded by Spike2 software (Cambridge Electronic Design Limited, Cambridge, UK). Signals were collected at a sampling frequency of 100 Hz and the software averaged every 10 samples to minimize fluctuations and noise. In order to deliver blue light to orexin-neurons, the silica fiber was implanted in the LHA.

At the start of the surgical procedures, *orexin-tTA* mice were anesthetized with isoflurane (2.5%, inhalation), and placed on a small animal stereotaxic frame (Model 963, David Kopf). Recombinant AAV-TetO (3G) G-CaMP6 (serotype: DJ, 600 nl/injection, 7 × 10^12^ copies/ml) was unilaterally injected into the LHA (from bregma −1.5 mm, lateral ± 0.8 mm, ventral −5.0 mm) with a glass micropipette and air pressure injector system (Pneumatic PicoPump; World Precision Instruments, Inc., Sarasota, FL, USA).

At least 3 weeks after viral injection, mice were surgically implanted with a silica fiber for recording of orexin neurons. The fiber was placed just above the LHA (from bregma −1.5 mm, lateral ± 0.8 mm, ventral −5.0 mm). Recording was started once the mice had recovered from anesthetization. Forceps with a force sensor (PIS-2001, manufactured by Aizawa S., Goto College of Medical Arts and Science, Tokyo, Japan) were used for mechanical stimulation. Pinch stimulation with the forceps was applied to the tail at forces of 100 g, 200 g and 300 g in either conscious mice or anesthetized mice. Additionally, a heating probe with a heat sensor was used for heat stimulation. A ramp-shaped noxious heat stimulus gradually increasing from 30 °C to 50 °C or 60 °C at 2 °C/sec was applied to the left hind-paw using a thermal stimulator with a circular probe (diameter: 1 mm, intercross-2000 N, Intercross, Tokyo, Japan). The probe was then cooled by circulating water, and this gradient of cooling was approximately 1.5 °C/sec.

### Drugs and pharmacogenetic drug administration

Clozapine N-oxide (CNO) was purchased from Enzo Life Sciences (Tokyo, Japan). CNO was dissolved in water as a 10 mg/ml stock solution, and diluted with saline to a 100 μg/ml solution before use. I.p. injection of CNO (1 mg/kg) was done at 12:00 during the light period (zeitgeber time = 4) to observe the maximum effects on wakefulness.

### Statistical analysis

Statistical analyses were performed using ORIGIN 9 software (LightStone). Simple comparisons of the means and SEM were performed by Student’s t-test. Multiple comparisons of the means and SEM were performed by one-way ANOVA analyses followed by Tukey’s test or Dunn’s multiple comparison test. A P value of less than 0.05 was considered significant in these studies.

## Additional Information

**How to cite this article**: Inutsuka, A. *et al*. The integrative role of orexin/hypocretin neurons in nociceptive perception and analgesic regulation. *Sci. Rep.* **6**, 29480; doi: 10.1038/srep29480 (2016).

## Supplementary Material

Supplementary Information
