# Supplementary material for: The integrative role of orexin/hypocretin neurons in nociceptive perception and analgesic regulation
Source: Sci Rep. 2016 Jul 7;6:29480. doi: 10.1038/srep29480 (PMC4935841; doi:10.1038/srep29480)
Supplement: Supplementary Information [file srep29480-s1.docx]

**The integrative role of orexin/hypocretin neurons in nociceptive perception and analgesic regulation**

Ayumu Inutsuka^1^, Akira Yamashita^1^, Srikanta Chowdhury^1^, Junichi Nakai^2, 3^, Masamichi Okura^2, 3^, Toru Taguchi^1^ and Akihiro Yamanaka^1^*

1, Department of Neuroscience II, Research Institute of Environmental Medicine, Nagoya University, Nagoya, 464-8601 Japan

2, Saitama University Graduate School of Science and Engineering, 255 Shimo-Okubo, Sakura-ku, Saitama City, Saitama, 338-8570 Japan

3, Saitama University Brain Science Institute, 255 Shimo-Okubo, Sakura-ku, Saitama City, Saitama, 338-8570 Japan

Correspondence:

Akihiro Yamanaka Ph.D.

Department of Neuroscience II, Research Institute of Environmental Medicine

Nagoya University, Nagoya 464-8601, Japan

Tel: +81-52-789-3861, Fax: +81-52-789-3889

E-mail: yamank@riem.nagoya-u.ac.jp


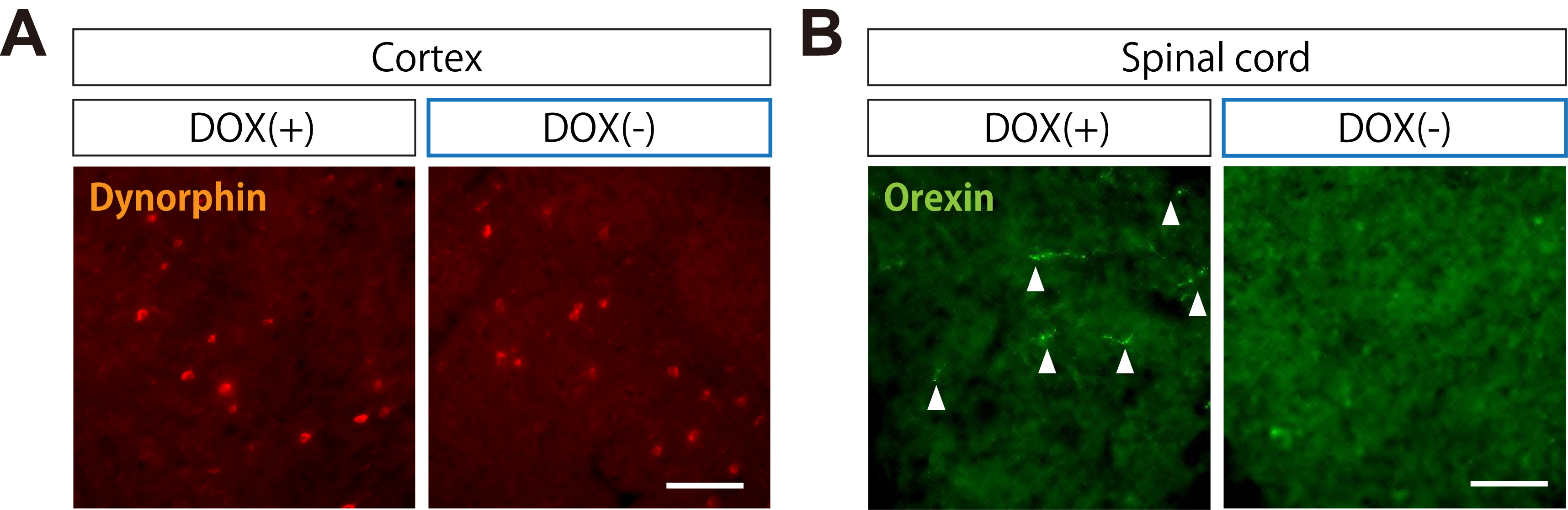
Supplementary Figure 1. Dynorphin-positive neurons in the cortex and orexin-positive axons in the spinal cord of *orexin-tTA; TetO DTA* mice

**A, B:** Coronal sections of the cortex (**A**) and the spinal cord (**B**) of *orexin-tTA; TetO DTA* mice fed with chow including DOX (DOX(+)) or chow without DOX (DOX(-)) for 4 weeks. The spinal cord at the L4 level includes nerve endings of orexin neurons in DOX(+) mice. However, these orexin nerve endings were not observed in DOX(-) mice.


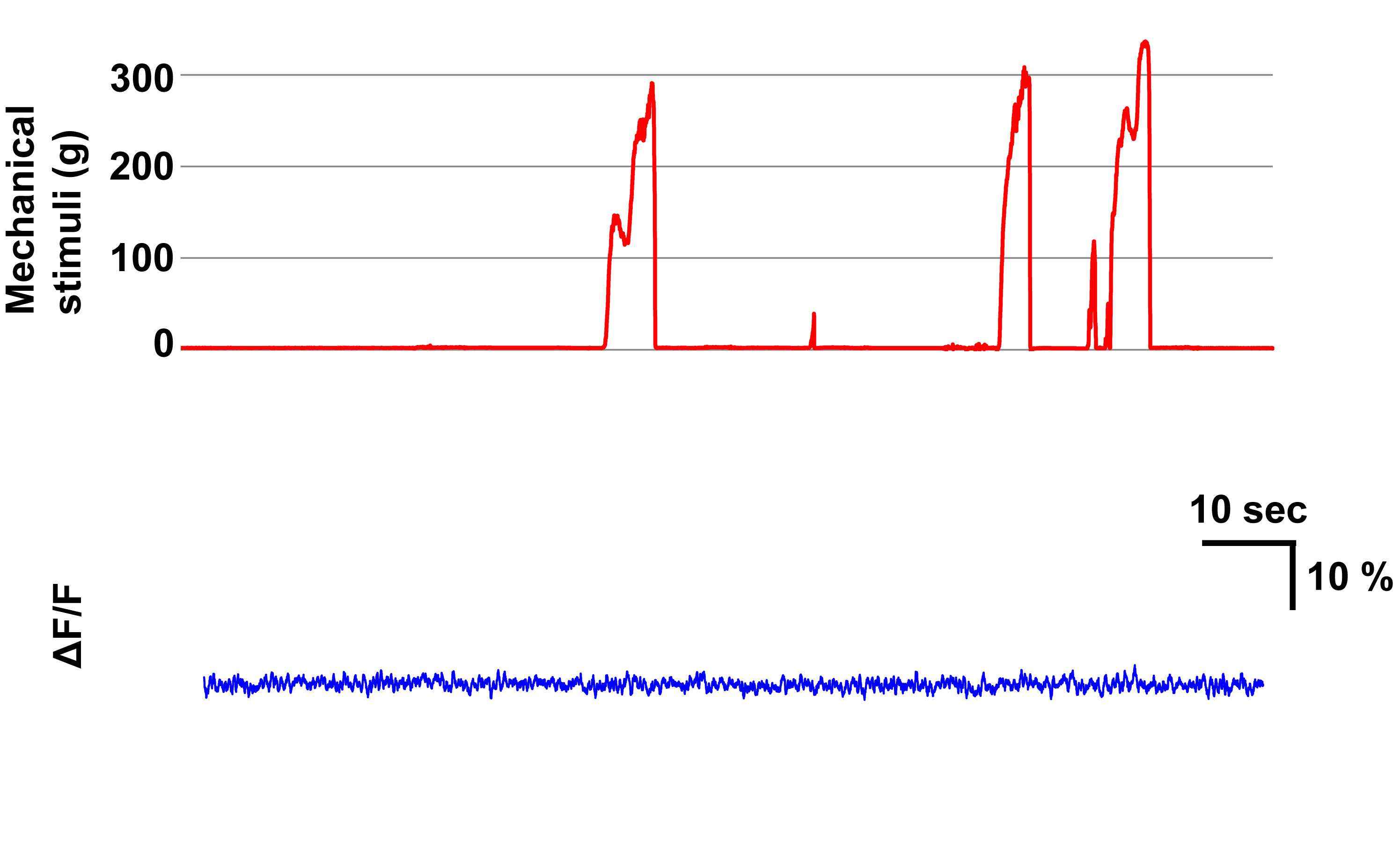
Supplementary Figure 2. Representative traces of fluorescence intensity in an orexin-EGFP mouse. Mechanical stimuli at 300 g did not affect the fluorescence intensity even when physical movement to avoid the pinching forceps was induced.
